# 1,3-Bis(4-meth­oxy­benz­yl)-6-methyl­pyrimidine-2,4(1*H*,3*H*)-dione

**DOI:** 10.1107/S1600536810024815

**Published:** 2010-06-30

**Authors:** Gong-Chun Li, Li-Ke Zhang, Zhi-Yu Ju, Feng-Ling Yang

**Affiliations:** a College of Chemistry and Chemical Engineering, Xuchang University, Xuchang, Henan Province 461000, People’s Republic of China

## Abstract

The title compound, C_21_H_22_N_2_O_4_, was prepared by reaction of 6-methyl­pyrimidine-2,4(1*H*,3*H*)-dione and 1-chloro­methyl-4-meth­oxy­benzene. In the title mol­ecule, the central pyrimidine ring forms dihedral angles of 62.16 (4) and 69.77 (3)° with the two benzene rings. In the crystal, weak inter­molecular C—H⋯O hydrogen bonds link the mol­ecules into chains.

## Related literature

For the applications of pyrimidine derivatives as pesticides and pharmaceutical agents, see: Condon *et al.* (1993 ▶); as agrochemicals, see: Maeno *et al.* (1990 ▶); as anti­viral agents, see: Gilchrist (1997 ▶); as herbicides, see: Selby *et al.* (2002 ▶); Zhu *et al.* (2007 ▶). For a related structure, see: Yang & Li (2006 ▶).
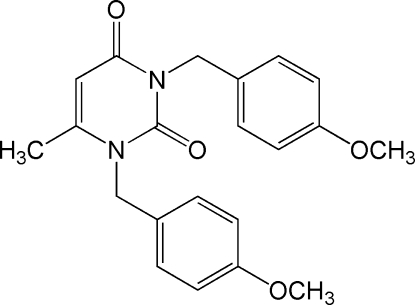


## Experimental

### 

#### Crystal data

C_21_H_22_N_2_O_4_
*M* *_r_* = 366.41
Monoclinic, 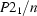

*a* = 8.4133 (9) Å
*b* = 9.929 (1) Å
*c* = 21.407 (3) Å
β = 91.614 (4)°
*V* = 1787.5 (3) Å^3^
*Z* = 4
Mo *K*α radiation
μ = 0.10 mm^−1^
*T* = 113 K
0.26 × 0.24 × 0.22 mm

#### Data collection

Rigaku Saturn724 CCD diffractometer
Absorption correction: multi-scan (*CrystalClear*; Rigaku/MSC, 2009 ▶) *T*_min_ = 0.976, *T*_max_ = 0.979
17394 measured reflections
4250 independent reflections
3035 reflections with *I* > 2σ(*I*)
*R*_int_ = 0.037

#### Refinement

*R*[*F* ^2^ > 2σ(*F* ^2^)] = 0.037
*wR*(*F* ^2^) = 0.096
*S* = 0.98
4250 reflections
247 parameters
H-atom parameters constrained
Δρ_max_ = 0.29 e Å^−3^
Δρ_min_ = −0.16 e Å^−3^

### 

Data collection: *CrystalClear* (Rigaku/MSC, 2009 ▶); cell refinement: *CrystalClear*; data reduction: *CrystalClear*; program(s) used to solve structure: *SHELXS97* (Sheldrick, 2008 ▶); program(s) used to refine structure: *SHELXL97* (Sheldrick, 2008 ▶); molecular graphics: *CrystalStructure* (Rigaku/MSC, 2009 ▶); software used to prepare material for publication: *CrystalStructure*.

## Supplementary Material

Crystal structure: contains datablocks global, I. DOI: 10.1107/S1600536810024815/pk2250sup1.cif

Structure factors: contains datablocks I. DOI: 10.1107/S1600536810024815/pk2250Isup2.hkl

Additional supplementary materials:  crystallographic information; 3D view; checkCIF report

## Figures and Tables

**Table 1:** Hydrogen-bond geometry (Å, °)

*D*—H⋯*A*	*D*—H	H⋯*A*	*D*⋯*A*	*D*—H⋯*A*
C20—H20⋯O3^i^	0.95	2.51	3.3627 (14)	150

Symmetry code: (i) 
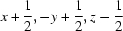
.

**Fig. 1.**
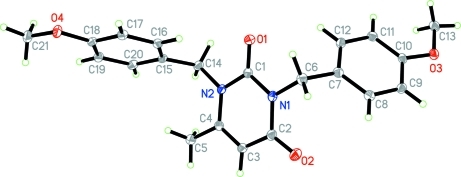
The asymmetric unit of the title compound, with displacement ellipsoids drawn at the 30% probability level.

**Fig. 2.**
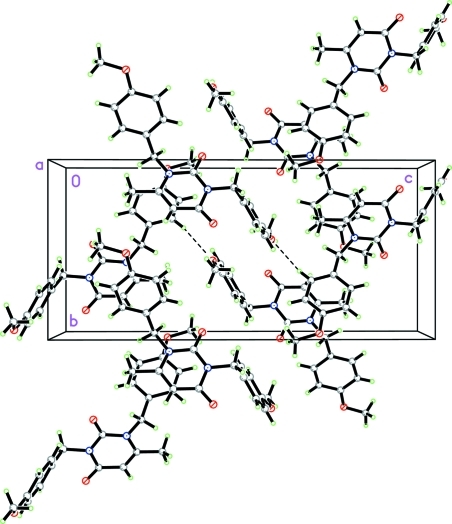
A packing diagram of the title compound viewed down the a axis. Intermolecular hydrogen bonds are shown as dashed lines.

## supplementary crystallographic information

### Comment

Pyrimidine derivatives are very important molecules in biology and have many applications in the areas of pesticide and pharmaceutical agents (Condon *et al.*, 1993). For example, imazosulfuron, ethirmol and mepanipyrim have been commercialized as agrochemicals (Maeno *et al.*, 1990). Pyrimidine derivatives have also been developed as antiviral agents, such as AZT, which is the most widely used anti-AIDS drug (Gilchrist, 1997). Recently, a new series of highly active herbicides of substituted pyrimidines were reported (Selby *et al.*, 2002); (Zhu *et al.*, 2007), and we have previously reported a related structure (Yang & Li 2006). As part of our goal to discover further biologically active pyrimidine compounds, the title compound was synthesized and its crystal structure determined (Fig. 1).

In the title molecule, the central pyrimidine ring forms dihedral angles of 62.16 (4) and 69.77 (3)° with the two benzene rings. In the crystal, weak intermolecular C—H···O hydrogen bonds link molecules.

### Experimental

6-methylpyrimidine-2,4(1*H*,3H)-dione (0.63 g, 5 mmol) and anhydrous potassium carbonate (0.84 g, 6 mmol) were mixed in dimethylformamide (20 ml). A solution of 1-(chloromethyl)-4-methoxybenzene (0.79 g, 5 mmol) in acetone (10 ml) was then added dropwise, with stirring at room temperature, and the mixture was stirred for another 10 h and then refluxed for 4 h. The solvent was evaporated *in vacuo* and the residue was washed with water. The resulting white precipitate was recrystallized from ethanol and single crystals were obtained by slow evaporation.

### Refinement

All H atoms were placed in calculated positions, with C—H = 0.95 Å, 0.98 Å or 0.99 Å, and included in the final cycles of refinement using a riding model, with *U*_iso_(H) = 1.2Ueq(C).

### Crystal data

**Table:**

C_21_H_22_N_2_O_4_	*F*(000) = 776
*M**_r_* = 366.41	*D*_x_ = 1.361 Mg m^−^^3^
Monoclinic, *P*2_1_/*n*	Mo *K*α radiation, λ = 0.71075 Å
Hall symbol: -P 2yn	Cell parameters from 5810 reflections
*a* = 8.4133 (9) Å	θ = 1.9–28.0°
*b* = 9.929 (1) Å	µ = 0.10 mm^−^^1^
*c* = 21.407 (3) Å	*T* = 113 K
β = 91.614 (4)°	Prism, colorless
*V* = 1787.5 (3) Å^3^	0.26 × 0.24 × 0.22 mm
*Z* = 4	

### Data collection

**Table:**

Rigaku Saturn724 CCD diffractometer	4250 independent reflections
Radiation source: fine-focus sealed tube	3035 reflections with *I* > 2σ(*I*)
graphite	*R*_int_ = 0.037
Detector resolution: 14.222 pixels mm^-1^	θ_max_ = 27.9°, θ_min_ = 1.9°
ω scans	*h* = −9→11
Absorption correction: multi-scan (*CrystalClear*; Rigaku/MSC, 2009)	*k* = −12→13
*T*_min_ = 0.976, *T*_max_ = 0.979	*l* = −28→28
17394 measured reflections	

### Refinement

**Table:**

Refinement on *F*^2^	Primary atom site location: structure-invariant direct methods
Least-squares matrix: full	Secondary atom site location: difference Fourier map
*R*[*F*^2^ > 2σ(*F*^2^)] = 0.037	Hydrogen site location: inferred from neighbouring sites
*wR*(*F*^2^) = 0.096	H-atom parameters constrained
*S* = 0.98	*w* = 1/[σ^2^(*F*_o_^2^) + (0.0556*P*)^2^] where *P* = (*F*_o_^2^ + 2*F*_c_^2^)/3
4250 reflections	(Δ/σ)_max_ = 0.001
247 parameters	Δρ_max_ = 0.28 e Å^−^^3^
0 restraints	Δρ_min_ = −0.16 e Å^−^^3^

### Special details

**Table:**

Geometry. All e.s.d.'s (except the e.s.d. in the dihedral angle between two l.s. planes) are estimated using the full covariance matrix. The cell e.s.d.'s are taken into account individually in the estimation of e.s.d.'s in distances, angles and torsion angles; correlations between e.s.d.'s in cell parameters are only used when they are defined by crystal symmetry. An approximate (isotropic) treatment of cell e.s.d.'s is used for estimating e.s.d.'s involving l.s. planes.
Refinement. Refinement of *F*^2^ against ALL reflections. The weighted *R*-factor *wR* and goodness of fit *S* are based on *F*^2^, conventional *R*-factors *R* are based on *F*, with *F* set to zero for negative *F*^2^. The threshold expression of *F*^2^ > σ(*F*^2^) is used only for calculating *R*-factors(gt) *etc*. and is not relevant to the choice of reflections for refinement. *R*-factors based on *F*^2^ are statistically about twice as large as those based on *F*, and *R*- factors based on ALL data will be even larger.

### Fractional atomic coordinates and isotropic or equivalent isotropic displacement parameters (Å^2^)

**Table:**

	*x*	*y*	*z*	*U*_iso_*/*U*_eq_	
O1	0.77270 (10)	−0.03179 (8)	0.39409 (4)	0.0326 (2)	
O2	1.05849 (10)	0.35414 (9)	0.42566 (4)	0.0349 (2)	
O3	0.38155 (9)	0.43800 (8)	0.57937 (3)	0.0258 (2)	
O4	1.17640 (10)	−0.48075 (8)	0.20574 (4)	0.0286 (2)	
N1	0.91553 (10)	0.16121 (9)	0.41019 (4)	0.0235 (2)	
N2	0.87518 (10)	0.07201 (9)	0.30901 (4)	0.0231 (2)	
C1	0.84910 (13)	0.06125 (12)	0.37267 (5)	0.0244 (3)	
C2	1.00559 (13)	0.27014 (12)	0.38848 (5)	0.0257 (3)	
C3	1.02826 (13)	0.27076 (12)	0.32258 (5)	0.0257 (3)	
H3	1.0893	0.3408	0.3049	0.031*	
C4	0.96571 (13)	0.17502 (11)	0.28489 (5)	0.0234 (2)	
C5	0.99072 (15)	0.17657 (13)	0.21620 (5)	0.0304 (3)	
H5A	1.0646	0.2492	0.2061	0.037*	
H5B	0.8888	0.1915	0.1940	0.037*	
H5C	1.0353	0.0900	0.2034	0.037*	
C6	0.89544 (14)	0.14901 (13)	0.47867 (5)	0.0275 (3)	
H6A	0.9940	0.1808	0.5003	0.033*	
H6B	0.8820	0.0526	0.4891	0.033*	
C7	0.75633 (13)	0.22666 (12)	0.50352 (5)	0.0229 (3)	
C8	0.77921 (13)	0.34760 (12)	0.53566 (5)	0.0244 (3)	
H8	0.8833	0.3838	0.5406	0.029*	
C9	0.65212 (13)	0.41528 (12)	0.56044 (5)	0.0240 (3)	
H9	0.6692	0.4972	0.5825	0.029*	
C10	0.49895 (13)	0.36351 (11)	0.55308 (5)	0.0213 (2)	
C11	0.47377 (14)	0.24358 (12)	0.52095 (5)	0.0250 (3)	
H11	0.3694	0.2080	0.5156	0.030*	
C12	0.60341 (13)	0.17605 (12)	0.49672 (5)	0.0248 (3)	
H12	0.5866	0.0935	0.4751	0.030*	
C13	0.22263 (13)	0.38888 (13)	0.57144 (6)	0.0292 (3)	
H13A	0.1932	0.3867	0.5268	0.035*	
H13B	0.1497	0.4485	0.5933	0.035*	
H13C	0.2160	0.2978	0.5888	0.035*	
C14	0.79638 (13)	−0.02957 (12)	0.26809 (6)	0.0263 (3)	
H14A	0.7576	0.0153	0.2293	0.032*	
H14B	0.7027	−0.0656	0.2895	0.032*	
C15	0.90264 (13)	−0.14602 (11)	0.25073 (5)	0.0224 (2)	
C16	0.97928 (13)	−0.22551 (12)	0.29611 (5)	0.0236 (3)	
H16	0.9686	−0.2036	0.3390	0.028*	
C17	1.06997 (13)	−0.33503 (12)	0.28015 (5)	0.0243 (3)	
H17	1.1212	−0.3876	0.3118	0.029*	
C18	1.08652 (13)	−0.36866 (11)	0.21741 (5)	0.0224 (2)	
C19	1.01334 (13)	−0.28976 (12)	0.17146 (5)	0.0251 (3)	
H19	1.0255	−0.3107	0.1286	0.030*	
C20	0.92206 (13)	−0.17982 (12)	0.18864 (5)	0.0249 (3)	
H20	0.8717	−0.1265	0.1570	0.030*	
C21	1.18686 (15)	−0.52057 (13)	0.14183 (5)	0.0324 (3)	
H21A	1.0798	−0.5359	0.1241	0.039*	
H21B	1.2489	−0.6038	0.1393	0.039*	
H21C	1.2391	−0.4493	0.1183	0.039*	

### Atomic displacement parameters (Å^2^)

**Table:**

	*U*^11^	*U*^22^	*U*^33^	*U*^12^	*U*^13^	*U*^23^
O1	0.0372 (5)	0.0225 (5)	0.0387 (5)	−0.0028 (4)	0.0125 (4)	−0.0013 (4)
O2	0.0361 (5)	0.0325 (5)	0.0360 (5)	−0.0066 (4)	−0.0008 (4)	−0.0107 (4)
O3	0.0232 (4)	0.0286 (5)	0.0256 (4)	0.0020 (3)	0.0020 (3)	−0.0037 (3)
O4	0.0340 (5)	0.0237 (5)	0.0280 (4)	0.0067 (4)	0.0025 (3)	−0.0041 (3)
N1	0.0242 (5)	0.0204 (5)	0.0259 (5)	0.0032 (4)	0.0031 (4)	−0.0023 (4)
N2	0.0226 (5)	0.0181 (5)	0.0287 (5)	0.0000 (4)	0.0040 (4)	−0.0044 (4)
C1	0.0224 (6)	0.0187 (6)	0.0322 (6)	0.0036 (5)	0.0054 (5)	−0.0008 (5)
C2	0.0213 (6)	0.0224 (6)	0.0334 (6)	0.0026 (5)	0.0004 (5)	−0.0041 (5)
C3	0.0240 (6)	0.0204 (6)	0.0328 (6)	−0.0003 (5)	0.0053 (5)	−0.0014 (5)
C4	0.0197 (6)	0.0202 (6)	0.0305 (6)	0.0034 (5)	0.0037 (4)	0.0000 (5)
C5	0.0354 (7)	0.0269 (7)	0.0292 (6)	−0.0024 (5)	0.0058 (5)	−0.0024 (5)
C6	0.0286 (6)	0.0266 (7)	0.0274 (6)	0.0056 (5)	0.0003 (5)	0.0015 (5)
C7	0.0267 (6)	0.0223 (6)	0.0197 (5)	0.0022 (5)	0.0012 (4)	0.0043 (4)
C8	0.0225 (6)	0.0276 (7)	0.0229 (6)	−0.0012 (5)	−0.0011 (4)	0.0016 (5)
C9	0.0285 (6)	0.0222 (6)	0.0212 (6)	−0.0012 (5)	−0.0009 (4)	−0.0016 (4)
C10	0.0252 (6)	0.0232 (6)	0.0158 (5)	0.0029 (5)	0.0022 (4)	0.0025 (4)
C11	0.0238 (6)	0.0279 (7)	0.0233 (6)	−0.0039 (5)	0.0012 (4)	0.0000 (5)
C12	0.0317 (6)	0.0214 (6)	0.0214 (5)	−0.0007 (5)	0.0024 (5)	−0.0005 (5)
C13	0.0234 (6)	0.0330 (7)	0.0316 (6)	0.0002 (5)	0.0049 (5)	0.0002 (5)
C14	0.0236 (6)	0.0220 (6)	0.0334 (6)	−0.0016 (5)	0.0003 (5)	−0.0049 (5)
C15	0.0196 (6)	0.0174 (6)	0.0303 (6)	−0.0033 (4)	0.0022 (4)	−0.0034 (5)
C16	0.0257 (6)	0.0228 (6)	0.0225 (5)	−0.0043 (5)	0.0036 (4)	−0.0023 (5)
C17	0.0264 (6)	0.0226 (6)	0.0237 (6)	−0.0017 (5)	−0.0008 (4)	0.0015 (5)
C18	0.0223 (6)	0.0173 (6)	0.0277 (6)	−0.0022 (5)	0.0027 (4)	−0.0023 (5)
C19	0.0301 (6)	0.0234 (6)	0.0217 (6)	−0.0025 (5)	0.0010 (4)	−0.0031 (5)
C20	0.0278 (6)	0.0209 (6)	0.0260 (6)	−0.0004 (5)	−0.0029 (4)	0.0016 (5)
C21	0.0394 (7)	0.0273 (7)	0.0309 (7)	0.0042 (6)	0.0067 (5)	−0.0069 (5)

### Geometric parameters (Å, °)

**Table:**

O1—C1	1.2220 (13)	C9—C10	1.3922 (15)
O2—C2	1.2276 (14)	C9—H9	0.9500
O3—C10	1.3677 (13)	C10—C11	1.3886 (16)
O3—C13	1.4288 (13)	C11—C12	1.3929 (15)
O4—C18	1.3723 (13)	C11—H11	0.9500
O4—C21	1.4292 (14)	C12—H12	0.9500
N1—C1	1.3848 (15)	C13—H13A	0.9800
N1—C2	1.4070 (15)	C13—H13B	0.9800
N1—C6	1.4853 (14)	C13—H13C	0.9800
N2—C4	1.3838 (14)	C14—C15	1.5142 (15)
N2—C1	1.3904 (14)	C14—H14A	0.9900
N2—C14	1.4803 (14)	C14—H14B	0.9900
C2—C3	1.4290 (15)	C15—C20	1.3850 (15)
C3—C4	1.3444 (16)	C15—C16	1.3954 (16)
C3—H3	0.9500	C16—C17	1.3770 (16)
C4—C5	1.4914 (15)	C16—H16	0.9500
C5—H5A	0.9800	C17—C18	1.3949 (15)
C5—H5B	0.9800	C17—H17	0.9500
C5—H5C	0.9800	C18—C19	1.3879 (16)
C6—C7	1.5108 (15)	C19—C20	1.3901 (16)
C6—H6A	0.9900	C19—H19	0.9500
C6—H6B	0.9900	C20—H20	0.9500
C7—C12	1.3850 (15)	C21—H21A	0.9800
C7—C8	1.3946 (16)	C21—H21B	0.9800
C8—C9	1.3814 (15)	C21—H21C	0.9800
C8—H8	0.9500		
			
C10—O3—C13	116.79 (9)	C11—C10—C9	120.04 (10)
C18—O4—C21	116.59 (9)	C10—C11—C12	119.15 (11)
C1—N1—C2	124.87 (9)	C10—C11—H11	120.4
C1—N1—C6	117.30 (9)	C12—C11—H11	120.4
C2—N1—C6	117.78 (9)	C7—C12—C11	121.40 (11)
C4—N2—C1	121.75 (9)	C7—C12—H12	119.3
C4—N2—C14	121.65 (9)	C11—C12—H12	119.3
C1—N2—C14	116.56 (9)	O3—C13—H13A	109.5
O1—C1—N1	122.15 (11)	O3—C13—H13B	109.5
O1—C1—N2	121.67 (10)	H13A—C13—H13B	109.5
N1—C1—N2	116.18 (10)	O3—C13—H13C	109.5
O2—C2—N1	119.80 (10)	H13A—C13—H13C	109.5
O2—C2—C3	125.52 (11)	H13B—C13—H13C	109.5
N1—C2—C3	114.68 (10)	N2—C14—C15	114.07 (9)
C4—C3—C2	121.90 (11)	N2—C14—H14A	108.7
C4—C3—H3	119.0	C15—C14—H14A	108.7
C2—C3—H3	119.0	N2—C14—H14B	108.7
C3—C4—N2	120.60 (10)	C15—C14—H14B	108.7
C3—C4—C5	121.37 (11)	H14A—C14—H14B	107.6
N2—C4—C5	118.03 (10)	C20—C15—C16	117.80 (10)
C4—C5—H5A	109.5	C20—C15—C14	120.46 (10)
C4—C5—H5B	109.5	C16—C15—C14	121.70 (10)
H5A—C5—H5B	109.5	C17—C16—C15	121.49 (10)
C4—C5—H5C	109.5	C17—C16—H16	119.3
H5A—C5—H5C	109.5	C15—C16—H16	119.3
H5B—C5—H5C	109.5	C16—C17—C18	119.94 (10)
N1—C6—C7	114.63 (9)	C16—C17—H17	120.0
N1—C6—H6A	108.6	C18—C17—H17	120.0
C7—C6—H6A	108.6	O4—C18—C19	124.37 (10)
N1—C6—H6B	108.6	O4—C18—C17	116.09 (10)
C7—C6—H6B	108.6	C19—C18—C17	119.54 (10)
H6A—C6—H6B	107.6	C18—C19—C20	119.54 (10)
C12—C7—C8	118.64 (10)	C18—C19—H19	120.2
C12—C7—C6	120.28 (11)	C20—C19—H19	120.2
C8—C7—C6	121.04 (10)	C15—C20—C19	121.68 (10)
C9—C8—C7	120.71 (11)	C15—C20—H20	119.2
C9—C8—H8	119.6	C19—C20—H20	119.2
C7—C8—H8	119.6	O4—C21—H21A	109.5
C8—C9—C10	120.06 (11)	O4—C21—H21B	109.5
C8—C9—H9	120.0	H21A—C21—H21B	109.5
C10—C9—H9	120.0	O4—C21—H21C	109.5
O3—C10—C11	124.45 (10)	H21A—C21—H21C	109.5
O3—C10—C9	115.51 (10)	H21B—C21—H21C	109.5
			
C2—N1—C1—O1	179.48 (10)	C7—C8—C9—C10	−0.49 (16)
C6—N1—C1—O1	2.11 (16)	C13—O3—C10—C11	−1.51 (15)
C2—N1—C1—N2	−0.05 (15)	C13—O3—C10—C9	178.71 (9)
C6—N1—C1—N2	−177.43 (9)	C8—C9—C10—O3	179.95 (9)
C4—N2—C1—O1	−178.01 (10)	C8—C9—C10—C11	0.15 (16)
C14—N2—C1—O1	4.12 (15)	O3—C10—C11—C12	−179.39 (10)
C4—N2—C1—N1	1.53 (15)	C9—C10—C11—C12	0.38 (16)
C14—N2—C1—N1	−176.34 (9)	C8—C7—C12—C11	0.28 (16)
C1—N1—C2—O2	179.50 (10)	C6—C7—C12—C11	177.94 (10)
C6—N1—C2—O2	−3.13 (15)	C10—C11—C12—C7	−0.60 (16)
C1—N1—C2—C3	−1.11 (15)	C4—N2—C14—C15	84.38 (13)
C6—N1—C2—C3	176.25 (9)	C1—N2—C14—C15	−97.75 (11)
O2—C2—C3—C4	−179.76 (12)	N2—C14—C15—C20	−126.06 (11)
N1—C2—C3—C4	0.89 (16)	N2—C14—C15—C16	56.27 (14)
C2—C3—C4—N2	0.48 (17)	C20—C15—C16—C17	−0.72 (16)
C2—C3—C4—C5	−179.79 (10)	C14—C15—C16—C17	177.00 (10)
C1—N2—C4—C3	−1.78 (16)	C15—C16—C17—C18	−0.11 (16)
C14—N2—C4—C3	175.98 (10)	C21—O4—C18—C19	−3.25 (15)
C1—N2—C4—C5	178.48 (10)	C21—O4—C18—C17	176.61 (10)
C14—N2—C4—C5	−3.75 (15)	C16—C17—C18—O4	−178.78 (9)
C1—N1—C6—C7	−95.32 (12)	C16—C17—C18—C19	1.09 (16)
C2—N1—C6—C7	87.11 (12)	O4—C18—C19—C20	178.63 (10)
N1—C6—C7—C12	78.75 (13)	C17—C18—C19—C20	−1.22 (16)
N1—C6—C7—C8	−103.65 (12)	C16—C15—C20—C19	0.58 (16)
C12—C7—C8—C9	0.27 (16)	C14—C15—C20—C19	−177.17 (10)
C6—C7—C8—C9	−177.37 (10)	C18—C19—C20—C15	0.38 (17)

### Hydrogen-bond geometry (Å, °)

**Table:**

*D*—H···*A*	*D*—H	H···*A*	*D*···*A*	*D*—H···*A*
C20—H20···O3^i^	0.95	2.51	3.3627 (14)	150

Symmetry codes: (i) *x*+1/2, −*y*+1/2, *z*−1/2.
